# Global prevalence and characteristics of non-suicidal self-injury between 2010 and 2021 among a non-clinical sample of adolescents: A meta-analysis

**DOI:** 10.3389/fpsyt.2022.912441

**Published:** 2022-08-10

**Authors:** Qingqing Xiao, Xiaozhen Song, Lijuan Huang, Dandan Hou, Xuehua Huang

**Affiliations:** ^1^Mental Health Center, West China Hospital, Sichuan University, Chengdu, China; ^2^West China School of Nursing, Sichuan University, Chengdu, China

**Keywords:** adolescents, non-suicidal self-injury, prevalence, characteristics, meta-analysis

## Abstract

**Background:**

Adolescents with immature mind and unstable emotional control are high-risk groups of non-suicidal self-injury (NSSI) behavior. We meta-analyzed the global prevalence of NSSI and prevalence of NSSI characteristics in a non-clinical sample of adolescents between 2010 and 2021.

**Methods:**

A systematic search for relevant articles published from January 1, 2010 to June 30, 2021 was performed within the scholarly database search engines of CBM, CNKI, VIP, Wanfang, PubMed, Web of Science, PsycINFO, and Embase. Eligibility criteria were as follows: provided cross-sectional data on the prevalence of NSSI; the subjects were non-clinical sample adolescents; and a clear definition of NSSI was reported. We used the following definiton of NSSI as our standard: the deliberate, self-inflicted destruction of body tissue, such as cutting, burning, and biting, without attempted suicide. The quality evaluation tool for cross-sectional studies recommended by the JBI was used. The global prevalence of NSSI was calculated based on the random-effects model by Comprehensive Meta-analysis version 3.0. Subgroup analyses were performed to compare the prevalence according to sex, living place, smoking or drinking history, and family structure.

**Results:**

Sixty-two studies involving 264,638 adolescents were included. The aggregate prevalence of NSSI among a non-clinical sample of adolescents was similar between over a lifetime (22.0%, 95% CI 17.9–26.6) and during a 12-month period (23.2%, 95% CI 20.2–26.5). Repetitive NSSI was more common than episodic NSSI (20.3% vs. 8.3%) but the frequency of mild injury (12.6%) was similar to that of moderate injury (11.6%). Multiple-method NSSI occurred slightly more often compared than one-method NSSI (16.0% vs. 11.1%). The top three types of NSSI in adolescents were banging/hitting (12.0%, 95% CI 8.9–15.9), pinching (10.0%, 95% CI 6.7–14.8), and pulling hair (9.8%, 95% CI 8.3–11.5), and the least common type was swallowing drugs/toxic substances/chemicals (1.0%, 95% CI 0.5–2.2). Subgroup analyses showed that being female, smoking, drinking, having siblings, and belonging to a single-parent family may be linked to higher prevalence of NSSI.

**Conclusion:**

This meta-analysis found a high prevalence of NSSI in non-clinical sample of adolescents, but there are some changes in severity, methods, and reasons. Based on the current evidence, adolescents in modern society are more inclined to implement NSSI behavior by a variety of ways, which usually are repetitive, and moderate and severe injuries are gradually increasing. It is also worth noting that adolescents with siblings or in single-parent families are relatively more likely to implement NSSI behavior due to maladjustment to the new family model. Future research needs to continue to elucidate the features and risk factors of NSSI so as to intervene in a targeted way.

**Limitation:**

The limitation of this study is that the heterogeneity among the included studies is not low, and it is mainly related to Chinese and English studies. The results of this study should be used with caution.

**Systematic review registration:**

[www.crd.york.ac.uk/prospero/], identifier [CRD42022283217].

## Introduction

Non-suicidal self-injury (NSSI) behavior in adolescents is an ongoing societal health concern and is defined as the deliberate, direct, and socially unacceptable destruction of body tissue, such as skin cutting, skin burning, and hitting oneself, but without an attempt at suicide (1, 2). The possible motivation and potential purpose of NSSI behavior in adolescents might be to remove difficulties in life, release pressure or control emotion (3). NSSI behavior often carries a high risk of personal injury and high risk of repetition, which can increase the occurrence of suicidal behavior and seriously endanger the physical and mental health of adolescents (4, 5). Many lines of evidence indicate that while adolescents are physically mature during puberty, they have yet to reach psychological maturity, have higher levels of impulsivity, and may experience difficulty in regulation of negative emotions and be prone to engage in NSSI behaviors (6). Moreover, NSSI during adolescence can have long-lasting and far-reaching developmental consequences, manifesting as anxiety, depression, and suicidal behaviors later in life as well as increased burden on society and families (7). The prevalence of NSSI in adolescents increased significantly at the beginning of the 21st century, and the incidence remains high (8).

In China, a total of 15,623 adolescents in rural regions were engaged in a nationwide survey by using a multistage sampling method, and approximately 29% of them reported a history of NSSI at least once during the last year (9). In the United States, a 2015 survey by the Centers for Disease Control and Prevention Youth Risk Behavior Surveillance System estimated the prevalence of NSSI behavior among high-school-age adolescents (*n* = 64671) in 11 US states. It concluded that 6.4–30.8% of adolescents had purposefully engaged in NSSI behavior without attempted suicide during the past 12 months (10). A cross-sectional assessment comprising 12,068 adolescents in 11 European countries determined the lifetime prevalence of direct self-injurious behavior (D-SIB) to be 27.6%, corresponding to 19.7% for occasional D-SIB and 7.8% for repetitive D-SIB. Lifetime prevalence varied from 17.1 to 38.6% across countries (11). According to a meta-analysis, the average lifetime prevalence of primary occurrence of NSSI in school-aged adolescents worldwide was 17.2% (range 8.0–26.3%) (12). Another meta-analysis involving 686,672 children and adolescents found a 22.1% (95% CI 16.9–28.4) lifetime prevalence of NSSI and 19.5% (95% CI 13.3–27.6) in a 12-month time period (13). It is not difficult to see that NSSI has become one of the key health problems in the field of adolescent psychology in the past decade. However, the epidemic characteristics and influencing factors of NSSI in different regions of the world are quite different.

Therefore, we conducted a meta-analysis to estimate the global prevalence of NSSI behavior and research its characteristics in adolescents. In this context, we were able to identify epidemiological and social factors associated with NSSI that could be used to deliver timely assistance and intervention in the future.

## Methods

This study was conducted by following the Preferred Reporting Items for Systematic Reviews and Meta-Analyses (PRISMA) (14), with the registration number of CRD42022283217 on PROSPERO.

### Search strategy and eligibility criteria

A systematic search within the literature was performed using the electronic databases China Biological Medicine (CBM), China National Knowledge Infrastructure (CNKI), VIP database, Wanfang database, PubMed, Web of Science, PsycINFO, and Embase, from January 1, 2010 to June 30, 2021. In this study, we use the combination of Mesh words and free words for literature search. The following search terms or combination thereof were used (* indicates truncation): (“self-harm” or “self-injury”) and (“adolescent” or “youth” or “young” or “teen*” or “student*” or “school*”) and (“prevalence”). Reference lists from the retrieved literature were also examined to identify additional studies.

Two authors (X-zS and L-jH) independently confirmed the eligibility of studies by screening title and abstract. Studies published in English or Chinese were considered. Any dissonance between the two authors was communicated and jointly resolved. Eligibility criteria are as follows: provided cross-sectional data on the prevalence of NSSI; the subjects are non-clinical sample adolescents who are those between the ages of 10 and 19; and a clear definition of NSSI was reported. We used the following definiton of NSSI as our standard: the deliberate, self-inflicted destruction of body tissue, such as cutting, burning, and biting, without attempted suicide (1, 2). Any study that did not meet the above inclusion criteria was excluded.

### Data extraction

Two authors (L-jH and D-dH) independently and manually extracted data from eligible studies after reading the full-length text. The following data were extracted: name of first author, year of publication, country of origin, study design, instrument for NSSI assessment, participant gender, total sample size, mean age of participants, and prevalence of NSSI. Prevalence of NSSI was considered our primary outcome. Disagreements about data extraction were resolved by the corresponding author (X-hH). We used the quality evaluation tool for cross-sectional studies recommended by the Joanna Briggs Institute (JBI) (15).

### Statistical analysis

All statistical analyses were conducted with Comprehensive Meta-analysis version 3.0. The *I*^2^ statistic was used to assess the between-study heterogeneity, which described the percentage of variance on a basis of real differences in study effects. An *I*^2^ value of 25% was considered low, 50% moderate and 75% substantial. If significant heterogeneity was detected, the random-effects model was applied. The random-effects model assumes various effect sizes between studies, different study designs and study subjects. Thus, the aggregate prevalence of NSSI was calculated based on the random-effects model, and data were reported with the corresponding 95% confidence interval (CI) where appropriate. The statistical significance level was set at *p* < 0.05.

Publication bias was assessed using the funnel plot along with Egger’s and Begg’s tests. A *p* value of 0.05 or less was used as the cut off for the presence of statistically significant publication bias. Subgroup analyses were performed to compare the aggregate prevalence of NSSI outcome in each study as a function of sex, living place, smoking, or drinking history, and family structure. Sensitivity analyses were performed by changing the combined effect model to explore potential sources of heterogeneity.

## Results

### Study selection and characteristics

**FIGURE 1:**
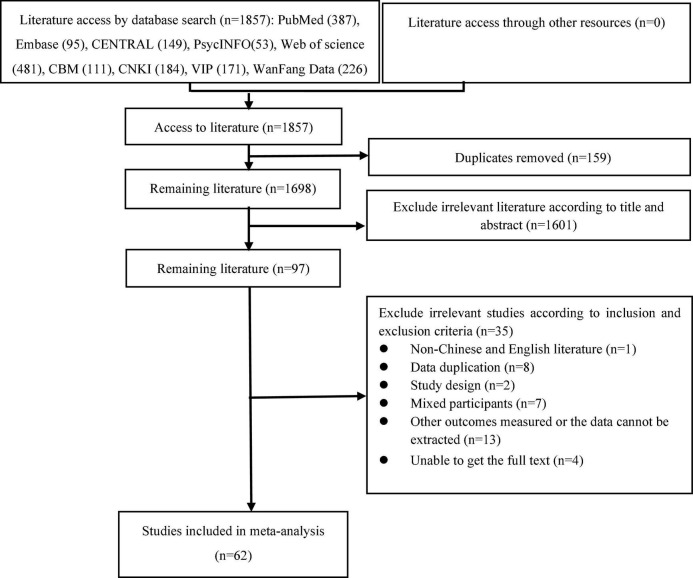
Flow chart of literature search.

The detailed process of paper selection is displayed in Figure 1. A total of 1,857 relevant citations were gathered after an extensive literature search was performed in several databases. Duplicates (*n* = 159) were removed, and a screen of titles and abstracts determined that an additional 1,601 were irrelevant. The resulting 97 studies were comprehensively reviewed, and an additional 35 were excluded. Finally, 62 studies including 264,638 subjects were used in this meta-analysis.

Characteristics of the included studies are shown in Table 1.

**TABLE 1:** Characteristics of the included studies.

Study	Country of origin	Instrument for NSSI assessment	Sample size			Mean age	Prevalence of NSSI, %	
			Male	Female	Total		Past year	Lifetime
Yan et al., 2012 (16)	China	RBQ-A	705	583	1288	14.24	22.67	NA
Giletta et al., 2012 (17)	Italy; Netherlands	6-item measure	NA	NA	1502	15.69	22.84	NA
Di Pierro et al., 2012 (18)	Italy	SIQ-TR	79	188	267	17.03	13.48	18.4
Sornberger et al., 2012 (19)	United States	Single-item measure	3503	3623	7126	14.92	NA	24.47
Tang et al., 2013 (20)	China	FASM	1436	1471	2907	15.4	33.6	NA
Tormoen et al., 2013 (21)	United States	Single-item measure	NA	NA	11440	NA	NA	4.3
Cheung et al., 2013 (22)	China	Single-item measure	1047	1270	2317	16.4	13.98	NA
Zetterqvist et al., 2013 (23)	Sweden	FASM	1515	1545	3060	NA	35.6	41.6
Liang et al., 2014 (24)	China	8-item measure	1089	1031	2140	14	NA	23.1
Rodav et al., 2014 (25)	Israel	OSI-F	NA	NA	275	14.81	20.7	NA
Liang et al., 2014 (26)	China	SHQ	1085	1046	2131	13.92	NA	23.2
Evren et al., 2014 (27)	Turkey	Single-item measure	NA	NA	4957	15.58	14.4	NA
Albores-Gallo et al., 2014 (28)	Mexico	Self-injury questionnaire	244	289	533	13.37	12.6	17.1
Claes et al., 2014 (29)	Belgium	SHI	395	137	532	15.11	NA	26.5
Claes et al., 2015 (30)	Belgium; Netherlands	SHI	436	349	785	15.56	NA	20.1
Hanania et al., 2015 (31)	Jordan	Single-item measure	478	474	952	NA	14.29	22.6
Kiekens, 2015 (32)	Belgium; Netherlands	SHI	511	408	946	15.52	NA	24.31
Gandhi et al., 2015 (33)	Belgium	SIQTR	201	335	568	16.13	NA	16.5
Calvete et al., 2015 (34)	Spain	FASM	901	959	1864	15.32	32.2	NA
Somer et al., 2015 (35)	Turkey	ISAS	745	911	1656	16.8	NA	31.3
Kim and Yu, 2017 (36)	South Korea	DSHI	376	341	717	NA	NA	8.8
Cimen et al., 2017 (37)	Turkey	ISAS	241	314	555	NA	NA	11.4
Liu et al., 2017 (38)	China	Single-item measure	1027	1063	2090	15.5	12.6	8.8
Lin et al., 2017 (39)	China	Twelve NSSI behaviors	1007	1108	2161	15.83	20.1	NA
Ma et al., 2018 (40)	China	Adolescent NSSI behavior questionnaire	4600	5104	9704	NA	38.50	NA
Jiang et al., 2018 (41)	China	Chinese version of YRBSS	1005	805	1910	NA	6.80	NA
Cui et al., 2018 (42)	China	Single-item measure	2033	1704	3737	NA	34.7	NA
Gandhi et al., 2018 (43)	Belgium	Single-item measure	NA	NA	401	16.6	NA	16.5
Liu et al., 2018 (44)	China	Single-item measure	NA	NA	5696	15.0	21.4	28.1
Tang et al., 2018 (9)	China	Chinese-FASM	8043	7580	15623	15.2	29.2	NA
Ren et al., 2018 (45)	China	DSHI	955	1034	1989	15.45	20.8	NA
Jiang et al., 2018 (46)	China	DSHI	579	447	1026	13.76	24.2	NA
Cao et al., 2019 (47)	China	Single-item measure	1075	1029	2104	NA	NA	10.9
Chen et al., 2019 (48)	China	OSI	4150	2979	7129	15.48	NA	33.7
Chen et al., 2019 (49)	China	8-item measure	7250	6192	14162	15.13	15.36	NA
Ma et al., 2019 (50)	China	8-item measure	7999	7539	15538	15.13	28.74	NA
Xu et al., 2019 (51)	China	ANSAQ	10862	10969	21831	15	7.9	NA
Zhang and Zhang, 2019 (52)	China	Adolescents’ non-suicidal self-injury scale	708	789	1497	12.01	NA	9.9
Li et al., 2019 (53)	China	8-item measure	10990	11638	22628	15.36	32.1	NA
Gaspar et al., 2019 (54)	Portugal	Single-item measure	1499	1763	3262	14.8	20.3	NA
Hu et al., 2020 (55)	China	OSI	4150	2979	7129	15.48	33.7	NA
Hu et al., 2020 (56)	China	ANSAQ	3995	3130	7125	13.93	51.40	NA
Jiang et al., 2020 (57)	China	ANSAQ	7347	7153	14500	14.83	14.81	NA
Lin et al., 2020 (58)	China	Modified Adolescents’ Self-Harm Scale	997	1068	2065	NA	NA	40.34
Mao et al., 2020 (59)	China	Modified Adolescents’ Self-Harm Scale	308	333	641	16.37	NA	32.1
Pang and Wang, 2020 (60)	China	Self injury behavior assessment questionnaire	7648	7174	14822	15.27	30.54	NA
Wang et al., 2020 (61)	China	Fourteen NSSI behaviors	412	363	775	15.58	41.3	NA
Zhou et al., 2020 (62)	China	OSI	2219	2215	4434	14.38	33.3	NA
Liu et al., 2020 (63)	China	Adolescent NSSI Function Assessment Scale	1245	1460	2705	13.4	NA	47.1
Tang et al., 2020 (64)	China	Chinese-FASM	8043	7580	15623	15.1	28.58	NA
Gu et al., 2020 (65)	China	Seven NSSI behaviors	NA	NA	949	13.35	38.9	NA
Buelens et al., 2020 (66)	Belgium	Single-item measure	NA	NA	2130	15	NA	21.8
Liang et al., 2021 (67)	China	DSHI	670	611	1281	10.60	NA	42.31
Sun et al., 2021 (68)	China	RBQ-A	534	466	1000	NA	NA	27.6
Costa et al., 2021 (69)	Brazil	FASM	254	251	505	14.32	45.3	NA
Perez et al., 2021 (70)	Spain	ISAS	809	924	1733	15.76	NA	24.6
Madjar et al., 2021 (71)	Israel	NSSI-AT	148	158	306	NA	11.4	NA
Jeong and Kim, 2021 (72)	South Korea	Single-item measure	968	879	1843	NA	8.8	NA
Lee et al., 2021 (73)	South Korea	Korean-DSHI	1075	599	1674	16.6	28.3	NA
Tang et al., 2021 (74)	China	Twelve NSSI behaviors	545	504	1060	14.66	40.9	NA
Jiang et al., 2021 (75)	China	Seven NSSI behaviors	356	372	728	14.07	17.4	NA
Abbasian et al., 2021 (76)	Iran	ISAS	NA	NA	604	14.29	NA	38.7

SHQ, self-harm questionnaire; RBQ-A, risky behavior questionnaire for adolescents; YRBSS, youth risk behavior surveillance system; OSI, Ottawa self-injury; ANSAQ, adolescent non-suicidal self-injury assessment questionnaire; DSHI, deliberate self-harm inventory; SIQTR, self-injury questionnaire-treatment related; FASM, functional assessment of self-mutilation; OSI-F, Ottawa self-injury inventory-functions; SHI, self-harm inventory; ISAS, inventory of statements about self-injury; NSSI-AT, non-suicidal self-injury assessment tool; NA, not available.

### Quality assessment of included studies

Most of the included studies (44, 71%) were of high quality, complied with all items of the quality evaluation tool for cross-sectional studies recommended by the JBI, but a few included studies (18, 29%) did not clearly give the content required for evaluation (Table 2).

**TABLE 2:** Quality assessment of included studies.

Study	Q1^a^	Q2^a^	Q3^a^	Q4^a^	Q5^a^	Q6^a^	Q7^a^	Q8^a^	Q9^a^
Yan et al., 2012 (16)	Yes	Yes	Yes	Yes	Yes	Yes	Yes	Yes	Yes
Giletta et al., 2012 (17)	Yes	Unclear	Yes	Yes	Yes	Yes	Yes	Yes	Yes
Di Pierro et al., 2012 (18)	Yes	Unclear	No	Yes	Yes	Yes	Yes	Yes	Unclear
Sornberger et al., 2012 (19)	Yes	Yes	Yes	Yes	Yes	Yes	Yes	Yes	Yes
Tang et al., 2013 (20)	Yes	Yes	Yes	Yes	Yes	Yes	Yes	Yes	Yes
Tormoen et al., 2013 (21)	Yes	Yes	Yes	Yes	Yes	No	Yes	Yes	Yes
Cheung et al., 2013 (22)	Yes	Yes	Yes	Yes	Yes	Yes	Yes	Yes	Yes
Zetterqvist et al., 2013 (23)	Yes	Yes	Yes	Yes	Yes	Yes	Yes	Yes	Yes
Liang et al., 2014 (24)	Yes	Yes	Yes	Yes	Yes	Yes	Yes	Yes	Yes
Rodav et al., 2014 (25)	Yes	Yes	No	Yes	Yes	Yes	Yes	Yes	Unclear
Liang et al., 2014 (26)	Yes	Yes	Yes	Yes	Yes	Yes	Yes	Yes	Yes
Evren et al., 2014 (27)	Yes	Yes	Yes	Yes	Yes	Yes	Yes	Yes	Yes
Albores-Gallo et al., 2014 (28)	Yes	No	Yes	Yes	Yes	Yes	Yes	Yes	Unclear
Claes et al., 2014 (29)	Yes	Unclear	Yes	Yes	Yes	Yes	Yes	Yes	Unclear
Claes et al., 2015 (30)	Yes	No	Yes	Yes	Yes	Yes	Yes	Yes	Yes
Hanania et al., 2015 (31)	Yes	Unclear	Yes	Yes	Yes	Yes	Yes	Yes	Yes
Kiekens et al., 2015 (32)	Yes	Yes	Yes	Yes	Yes	Yes	Yes	Yes	Yes
Gandhi et al., 2015 (33)	Yes	Yes	Yes	Yes	Yes	Yes	Yes	Yes	Yes
Calvete et al., 2015 (34)	Yes	Yes	Yes	Yes	Yes	Yes	Yes	Yes	Yes
Somer et al., 2015 (35)	Yes	Yes	Yes	Yes	Yes	Yes	Yes	Yes	Yes
Kim and Yu, 2017 (36)	Yes	Unclear	Yes	Yes	Yes	Yes	Yes	Yes	Yes
Cimen et al., 2017 (37)	Yes	Unclear	Yes	Yes	Yes	Yes	Yes	Yes	Yes
Liu et al., 2017 (38)	Yes	Yes	Yes	Yes	Yes	Yes	Yes	Yes	Yes
Lin et al., 2017 (39)	Yes	Yes	Yes	Yes	Yes	Yes	Yes	Yes	Yes
Ma et al., 2018 (40)	Yes	Yes	Yes	Yes	Yes	Yes	Yes	Yes	Yes
Jiang et al., 2018 (41)	Yes	Yes	Yes	Yes	Yes	Yes	Yes	Yes	Yes
Cui et al., 2018 (42)	Yes	Yes	Yes	Yes	Yes	Yes	Yes	Yes	Yes
Gandhi et al., 2018 (43)	Yes	Yes	Unclear	Yes	No	Yes	Yes	Yes	Unclear
Liu et al., 2018 (44)	Yes	Yes	Yes	Yes	Yes	Yes	Yes	Yes	Yes
Tang et al., 2018 (9)	Yes	Yes	Yes	Yes	Yes	Yes	Yes	Yes	Yes
Ren et al., 2018 (45)	Yes	Yes	Yes	Yes	Yes	Yes	Yes	Yes	Yes
Jiang et al., 2018 (46)	Unclear	Unclear	Yes	Yes	Yes	Yes	Yes	Yes	Yes
Cao et al., 2019 (47)	Yes	Yes	Yes	Yes	Yes	Yes	Yes	Yes	Yes
Chen et al., 2019 (48)	Yes	Yes	Yes	Yes	Yes	Yes	Yes	Yes	Yes
Chen et al., 2019 (49)	Yes	Yes	Yes	Yes	Yes	Yes	Yes	Yes	Yes
Ma et al., 2019 (50)	Yes	Yes	Yes	Yes	Yes	Yes	Yes	Yes	Yes
Xu et al., 2019 (51)	Yes	Yes	Yes	Yes	Yes	Yes	Yes	Yes	Yes
Zhang and Zhang, 2019 (52)	Yes	Yes	Yes	Yes	Yes	Yes	Yes	Yes	Yes
Li et al., 2019 (53)	Yes	Yes	Yes	Yes	Yes	Yes	Yes	Yes	Yes
Gaspar et al., 2019 (54)	Yes	Yes	Yes	Yes	Yes	Yes	Yes	Yes	Yes
Hu et al., 2020 (55)	Yes	Yes	Yes	Yes	Yes	Yes	Yes	Yes	Yes
Hu et al., 2020 (56)	Yes	Yes	Yes	Yes	Yes	Yes	Yes	Yes	Yes
Jiang et al., 2020 (57)	Yes	Yes	Yes	Yes	Yes	Yes	Yes	Yes	Yes
Lin et al., 2020 (58)	Yes	Yes	Yes	Yes	Yes	Yes	Yes	Yes	Yes
Mao et al., 2020 (59)	Yes	Yes	Unclear	Yes	Yes	Yes	Yes	Yes	Yes
Pang and Wang, 2020 (60)	Yes	Yes	Yes	Yes	Yes	Yes	Yes	Yes	Yes
Wang et al., 2020 (61)	Yes	Yes	Unclear	Yes	Yes	Yes	Yes	Yes	Yes
Zhou et al., 2020 (62)	Yes	Yes	Yes	Yes	Yes	Yes	Yes	Yes	Yes
Liu et al., 2020 (63)	Yes	Yes	Yes	Yes	Yes	Yes	Yes	Yes	Yes
Tang et al., 2020 (64)	Yes	Yes	Yes	Yes	Yes	Yes	Yes	Yes	Yes
Gu et al., 2020 (65)	Yes	Yes	Yes	Yes	Yes	Yes	Yes	Yes	Yes
Buelens et al., 2020 (66)	Yes	Yes	Yes	Yes	Yes	Yes	Yes	Yes	Yes
Liang et al., 2021 (67)	Yes	Yes	Yes	Yes	Yes	Yes	Yes	Yes	Yes
Sun et al., 2021 (68)	Yes	Yes	Yes	Yes	Yes	Yes	Yes	Yes	Yes
Costa et al., 2021 (69)	Yes	Yes	Yes	Yes	Yes	Yes	Yes	Yes	Yes
Perez et al., 2021 (70)	Unclear	Unclear	Yes	Yes	Yes	Yes	Yes	Yes	Yes
Madjar et al., 2021 (71)	Yes	Yes	No	Yes	Yes	Yes	Yes	Yes	Yes
Jeong and Kim, 2021 (72)	Yes	Yes	Yes	Yes	Yes	Yes	Yes	Yes	Yes
Lee et al., 2021 (73)	Unclear	Unclear	Yes	Yes	Yes	Yes	Yes	Yes	Yes
Tang et al., 2021 (74)	Yes	Yes	Yes	Yes	Yes	Yes	Yes	Yes	Yes
Jiang et al., 2021 (75)	Unclear	Unclear	Unclear	Yes	Yes	Yes	Yes	Yes	Yes
Abbasian, 2021 (76)	Yes	Yes	Yes	Yes	Yes	Yes	Yes	Yes	Yes

^a^Q1–Q9 based on the Joanna Briggs Institute Risk Assessment (15).

### Aggregate prevalence of non-suicidal self-injury in adolescents

#### Lifetime and 12-month prevalence

**FIGURE 2:**
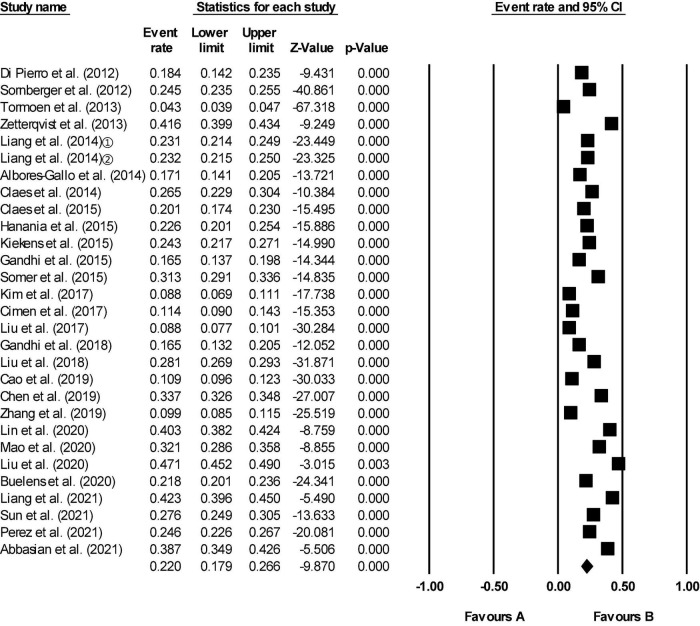
Forest plot of the lifetime aggregate prevalence of non-suicidal self-injury (NSSI) in adolescents. The location of the square represents the incidence of the event, the size of the square represents the weight, and the diamond represents the combined incidence.

**FIGURE 3:**
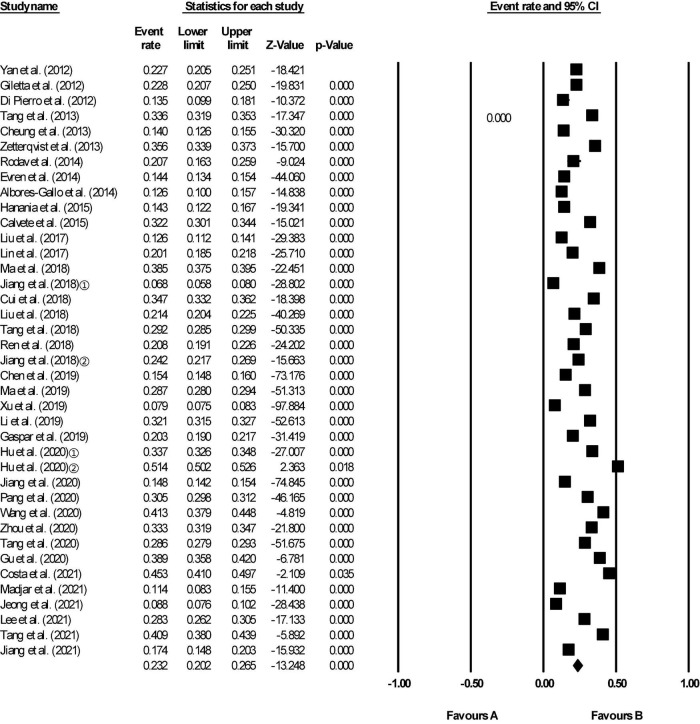
Forest plot of the 12-month aggregate prevalence of non-suicidal self-injury (NSSI) in adolescents.

Of the 62 included studies, some reported lifetime prevalence, some reported 12-month prevalence, and some both. In our study the lifetime aggregate prevalence of NSSI among 64,484 adolescents included in 29 studies was 22.0% (95% CI 17.9–26.6) (Figure 2). There was a significant level of heterogeneity detected (*I*^2^ = 99.393, *p* < 0.001). The 12-month aggregate prevalence of NSSI was only slightly higher when assessed in 39 studies (23.2%, 95% CI 20.2–26.5) involving a total of 212,752 adolescents (Figure 3). The heterogeneity remained significantly high with the additional studies (*I*^2^ = 99.660, *p* < 0.001).

### Aggregate prevalence of different characteristics of non-suicidal self-injury in adolescents

#### Frequency

Table 3 shows that the aggregate prevalence of episodic NSSI in adolescents was 8.3% (95% CI: 5.4–12.5), while 20.3% (95% CI 13.9–28.6) of adolescents reported repetitive NSSI.

**TABLE 3:** Prevalence of characteristics of non-suicidal self-injury in adolescents.

Characteristic	Number of studies (*n*)	NSSI prevalence (%)	95% CI	Heterogeneity test	
				*I*^2^/%	*p*
**Frequency**					
Episodic frequency	6	8.3	5.4–12.5	98.606	<0.001
Repetitive frequency	6	20.3	13.9–28.6	99.295	<0.001
**Severity**					
Minor/mild	5	12.6	6.4–23.3	99.432	<0.001
Moderate/severe	5	11.6	10.0–13.3	84.917	<0.001
**Method**					
One method	6	11.1	8.8–13.9	88.157	<0.001
Multiple methods	6	16.0	11.0–22.6	97.003	<0.001
**Type**					
Cutting	19	7.0	5.7–8.6	97.996	<0.001
Biting	12	8.6	6.4–11.4	98.957	<0.001
Burning	17	2.5	1.8–3.4	97.394	<0.001
Carving	7	7.8	5.1–12.0	97.608	<0.001
Pinching	4	10.0	6.7–14.8	96.367	<0.001
Pulling hair	10	9.8	8.3–11.5	97.429	<0.001
Scratching	13	8.6	6.6–10.9	97.755	<0.001
Banging/hitting	18	12.0	8.9–15.9	99.566	<0.001
Interfering with wounds	5	7.8	4.8–12.3	96.291	<0.001
Rubbing skin	3	3.6	2.0–6.6	96.620	<0.001
Sticking needles	3	3.6	1.8–7.0	96.664	<0.001
Swallowing drug/toxic substance/chemicals	3	1.0	0.5–2.2	93.874	<0.001

#### Severities

The aggregate prevalence of minor or mild NSSI in adolescents was 12.6% (95% CI 6.4–23.3), which was similar to that of moderate or severe NSSI (11.6%, 95% CI 10.0–13.3) (Table 3).

#### Method

One-method NSSI affected 11.1% (95% CI 8.8–13.9) of the adolescent population included in our meta-analysis (Table 3), with a slightly higher percentage reporting multiple-method NSSI (16.0%, 95% CI 11.0–22.6).

#### Type

The top three types of NSSI in adolescents were banging/hitting (12.0%, 95% CI 8.9–15.9), pinching (10.0%, 95% CI 6.7–14.8), and pulling hair (9.8%, 95% CI 8.3–11.5), and the least used type of self-harm was swallowing drugs/toxic substances/chemicals (1.0%, 95% CI 0.5–2.2) (Table 3).

### Subgroup analyses of non-suicidal self-injury among adolescents

#### Sex

When classified by gender, the prevalence of NSSI was significantly higher in females (25.4%, 95% CI 22.4–28.6) than in males (22.0%, 95% CI 19.2–25.0; *p* < 0.001) based on 43 studies (Table 4).

**TABLE 4:** Prevalence of non-suicidal self-injury among adolescents based on subgroup analyses.

Subgroup	Number of studies, *n*	Number of adolescents, *n*	NSSI prevalence, %	95% CI, %	Heterogeneity test		Subgroup differences			
					*I*^2^/%	*p*	*OR*	95% CI	*Z*	*p*
**Gender**										
Male	43	107,285	22.0	19.2–25.0	99.268	<0.001	0.839	0.768–0.918	–3.835	<0.001
Female	43	102,473	25.4	22.4–28.6	99.202	<0.001				
**Living place**										
Urban areas	10	37,514	26.6	20.6–33.5	99.428	<0.001	1.048	0.923–1.190	0.727	0.467
Rural areas	10	28,404	25.8	20.9–31.4	98.930	<0.001				
**Smoking history**										
Yes	3	1,479	24.7	12.4–43.1	93.050	<0.001	2.588	1.470–4.559	3.293	<0.001
No	3	4,072	10.1	3.2–27.6	99.149	<0.001				
**Drinking history**										
Yes	3	2,721	24.4	12.2–42.9	96.610	<0.001	3.014	1.487–6.108	3.060	0.002
No	3	2,850	9.3	3.1–24.8	98.677	<0.001				
**One child**										
Yes	16	49,014	25.8	22.5–29.3	98.611	<0.001	0.939	0.889–0.991	–2.269	0.023
No	16	86,402	27.0	24.0–30.3	99.077	<0.001				
**Single-parent family**										
Yes	4	1,203	30.1	27.6–32.8	1.758	0.383	1.200	1.056–1.363	2.379	0.017
No	4	19,959	23.5	19.0–28.5	97.183	<0.001				

#### Urban vs. rural

When the subjects in 10 studies were grouped by location, the prevalence of NSSI was found to be higher among adolescents living in urban areas (26.6%, 95% CI 20.6–33.5) than among those living in rural areas (25.8%, 95% CI 20.9–31.4), but this difference was not statistically significant (*p* > 0.05) (Table 4).

#### Smoking or drinking history

Prevalence of NSSI was significantly higher in adolescents who smoked (24.7%, 95% CI 12.4–43.1 vs. non-smoking: 10.1%, 95% CI 3.2–27.6, *p* < 0.01) and drank alcohol (24.4%, 95% CI 12.2–42.9 vs. non-drinking: 9.3%, 95% CI 3.1–24.8, *p* < 0.01). The results from three studies are shown in Table 4.

#### Family structure

Finally, NSSI was more prominent among adolescents in families with multiple children (27.0%, 95% CI 24.0–30.3) than among those in single-child families (25.8%, 95% CI 22.5–29.3). Moreover, the prevalence of NSSI was higher among adolescents in single-parent families (30.1%, 95% CI 27.6–32.8) than among those in two-parent families (23.5%, 95% CI 19.0–28.5). Differences were statistically significant in both scenarios (*p* < 0.05).

### Sensitivity analysis

In order to explore the stability of meta-analysis results, we repeated the meta-analysis with a fixed-effects model, which gave similar lifetime and 12-month aggregate prevalences of NSSI as the random-effects model. This suggested that our meta-analysis was reliable.

### Publication bias

**FIGURE 4:**
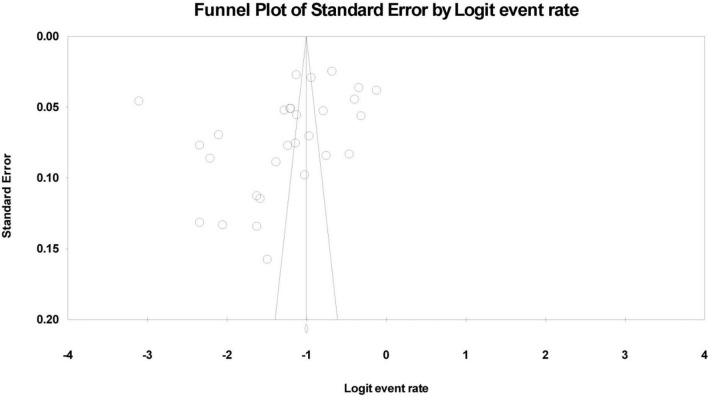
Funnel plot of the lifetime aggregate prevalence of non-suicidal self-injury (NSSI) in adolescents.

**FIGURE 5:**
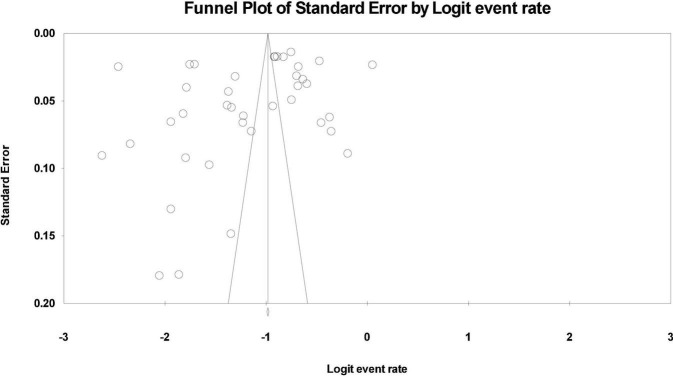
Funnel plot of the 12-month aggregate prevalence of non-suicidal self-injury (NSSI) in adolescents.

Asymmetry was detected in the funnel plot of the lifetime and 12-month aggregate prevalence rates (Figures 4, 5). Egger’s test showed no significant publication bias in the 29 studies (*t* = 1.97, *p* = 0.059) used to determine the lifetime rates, or in the 39 studies used to calculate the 12-month prevalence. However, the Begg’s test found significant publication bias within the studies used to calculate the lifetime aggregate prevalence (*Z* = 2.10, *p* = 0.035), but not in those studies referenced for the 12-month aggregate prevalence (*Z* = 1.68, *p* = 0.09).

## Discussion

Although NSSI in adolescents widespread, it is yet often a hidden problem. To the best of our knowledge, this is the first meta-analysis to study the global prevalence and characteristics of NSSI between 2010 and 2021 among a non-clinical sample of adolescents. This meta-analysis found a high prevalence of NSSI in adolescents. Repetitive NSSI was more common than episodic NSSI (20.3% vs. 8.3%) but the frequency of mild injury (12.6%) was similar to that of moderate injury (11.6%). Multiple-method NSSI occurred slightly more often compared than one-method NSSI (16.0% vs. 11.1%). The top three types of NSSI in adolescents were bang-ing/hitting, pinching, and pulling hair, and the least common type was swallowing drugs/toxic substances/chemicals. Subgroup analyses showed that being female, smoking, drinking, having siblings, and belonging to a single-parent family may be linked to higher prevalence of NSSI.

This study found that the aggregate prevalence rates were 22.0% during a lifetime and 23.2% during 12 months. This finding was consistent with the 22.1% lifetime prevalence of NSSI and 19.5% in a 12-month prevalence reported from a meta-analysis with 686,672 children and adolescents between 1989 and 2018 (13). Compared with that study, our study did not include children and focused on the prevalence of NSSI among adolescents in the last decade. It can be seen that the 12-month prevalence rate of NSSI was more higher in our study. However, it was lower than a comparative study done in 11 European countries among 12,068 adolescents showing lifetime prevalence varied from 17.1 to 38.6% (11). Still, our finding was higher than that another meta-analysis reported lifetime prevalence rate of NSSI in a worldwide was 17.2% (12). Despite these slight variations in findings, there is no doubt that the prevalence of NSSI is high worldwide. Adolescence is a sensitive and vulnerable period of time in which a person learns methods of internalizing and externalizing emotions, and a wide range of problematic behaviors can develop as a result of learning unhealthy coping mechanisms (77). Adolescents who have trouble expressing emotions and feelings may project a depressed mood characterized by impulsive and irritable self-injury and self-mutilation. Epidemiological investigation suggests that senior high school students with NSSI behavior often have seriously negative emotions and lack positive cognitive activities (78). When adolescents are in a stressful environment for a long time, or suddenly encounter a stressful event that exceeds their ability to cope, they may be attacked by negative emotions in the face of difficult situations that can not be easily solved, this in turn may induce impulsive and reckless behaviors. Sometimes, adolescents do express their feelings, parents often take a critical or neglectful attitude, which is more likely to lead to the child toward NSSI behavior (79). Other factors may also increase the likelihood of NSSI. For example, peer pressure may lead teenagers to self-mutilate in order to obtain a sense of identity and achievement. These same actions may also lead a teenager to feel embarrassment or inferiority to people around them. Oftentimes an adolescent may hide self-injury behavior and scars in order to avoid recalling the painful experience of the past (80). Schools should be made aware of the extent to which NSSI behavior is prevalent and problematic. This knowledge could guide the creation of safe environments where adolescents can go and learn how to deal with their emotions in positive ways, which could help prevent NSSI.

Our study found that adolescents were much more likely to injure themselves repeatedly by multiple methods, although the likelihood of mild or moderate injury seemed similar. This may reflect that self-injurious behavior can lead someone to feel that he or she is solving interpersonal problems, which may reduce negative thoughts or feelings, and instead generate positive emotions or feelings. To some extent, the more times an adolescent repeats the self-harm, the more they feel that they can control negative emotions. When these actions do not solve the actual problem, the risk of more severe consequences, such as suicide, are increased (81). The present study also found that the three most common types of NSSI in adolescents were banging/hitting, pinching, and pulling hair, while the least common type of NSSI in adolescents was swallowing drugs/toxic substances/chemicals. It is possible that adolescents rarely opt to swallow drugs/toxic substances/chemicals because of their preference for sensory stimulation: more physically involved attempts at self-harm may stimulate the senses more quickly and speed up the reactionary feeling of control. Although another study in 516 Korean adolescents found the incidence of cutting injury was high (19.3%) (82), the prevalence was only 7.0% in our meta-analysis. This may be related to the difficulty in acquiring dangerous goods in some countries, such as blades and sharp tools, or cutting injury was scary and bloody for most adolescents. Our results help to identify common types of self-injury and prevent possible self-injury.

Given that adolescence is a critical period to initiate self-injury prevention and intervention efforts (83), understanding the prevalence and features of NSSI is of great significance. Subgroup analyses showed that being female, smoking, drinking, having siblings, and being part of a single-parent family may increase risk of NSSI. According to our results, the prevalence of NSSI in female adolescents was higher than that in male adolescents. This was consistent with the research results in a study that NSSI showed to be associated with female gender (84). Female adolescents may be more susceptible to self-injury because they are more likely to experience higher negative influence and have lower ability to manage emotion, including acceptance of emotions and controlling impulses (78). Another study confirmed that menophania, irregular menstruation, and algomenorrhea were associated with an increased risk of NSSI (44). Smoking and drinking have also been positively associated with the prevalence of NSSI. Positive relationships of smoking, drinking, and self-injury with NSSI have also been reported in some previous studies (85–87). In addition, family structure and family ties may increase risk of NSSI. Our finding that adolescents from single-parent families were more prone to engage in self-injurious behavior was consistent with a study of Poland encompassed 5,685 individuals (88). It is possible that a connected family and solid parent-child ties can protect against self-injury (26). Research on the influence of familial ties on adolescent NSSI has thus far focused on the influence of parent-child relationships, while remarkably little is known about the influence of the relationships between relatives or between siblings. Our study found that adolescents with siblings were more likely to engage in self-injurious behavior than adolescents in single-child families. The bond between siblings is lifelong and represents one of the most important relationships in one’s life because children spend more time with their siblings than with their parents (89). The bond between siblings encompasses positive features (e.g., warmth, intimacy, empathy) but also negative features (e.g., conflict, rivalry), and it may have a major impact on each sibling’s life and wellbeing (90). Siblings may be a source of emotional support for each other (91). Our findings indicate that adolescents with siblings may face different peer interaction pressure, and may choose NSSI behavior as a signal to seek outside help in order to seek parental attention.

From the results of this study, we could see that in the 21st century, especially in the last decade, the incidence of adolescent NSSI behavior in non-clinical samples remains high, but there are some changes in severity, methods and reasons. Based on the current evidence, adolescents in modern society are more inclined to implement NSSI behavior by a variety of ways, which are repetitive and intentional, and moderate and severe injuries are gradually increasing. In terms of the types of NSSI, in the past, cutting was one of the main ways of self-injury, but the first three types of NSSI in this study were banging/hitting, pinching, and pulling hair. It is also worth noting that adolescents with siblings or single parent families are more prone to NSSI behavior. There may be three reasons as follows:

First, the temptation of virtual world and the influence of network environment on NSSI behavior. With the development of social economy and the popularity of new media on the internet, more and more adolescents are exposed to more complex and varied information about NSSI behavior on the internet. They will compare and discuss their own self-injury experience, and it is easier to try new ways of NSSI behavior (92).

Second, the increase of learning pressure, ineffective coping styles and out-of-control emotional self-management. Compared with the adolescents of the last century, the adolescents of the 21st century live in a more prosperous material environment. But facing a more intense competitive environment, they usually need not only learn the cultural knowledge of an age group, but also learn all kinds of talents or skills (93). When learning pressure is too high and the response is ineffective, their emotions are easy to get out of control, and they may have NSSI behaviors due to venting or avoiding bad emotions.

Third, adolescents’ interpersonal relationships are becoming more and more complex. Adolescents are gradually facing relatively complex peer relationships, teacher-student relationships, and family relationships. The instability of interpersonal relationship is easy to lead to cognitive deviation, negative emotions and problematic behaviors (92). Especially in China, with the opening of the comprehensive two-child policy, adolescents who used to be only children have a brother or sister with a large age difference, and the focus of the family has shifted away from themselves. When they feel helpless and have no help, NSSI behavior may become the last way to deal with it, because the visual impact of self-injury and the signal to the outside world are telling others that “I need help,” at the same time, it can also force others to respond, such as attracting the attention of parents (90). In addition, with the increasingly inclusive society, the increase of personal freedom and the improvement of marital autonomy, the divorce rate in contemporary society is much higher than that in the last century (94). Therefore, the number of children in single parent families is gradually increasing. With the change of family structure, family atmosphere and parental rearing patterns, adolescents are not easy to adapt to new family relationships and induce bad emotions and behaviors (88).

This study has several advantages. First, the meta-analysis shows minimal publication bias. Second, the aggregate prevalence of NSSI in adolescents was broken down in terms of frequencies, severities, methods, and types. Our findings contribute to raising awareness that NSSI in adolescents is a prevalent and unaddressed issue and should be addressed urgently. On the other hand, we acknowledge the following limitations in our study. First, all the included studies were in Chinese or English, so language bias cannot be ruled out. It is not difficult to find that more than half of the research comes from China. There may be two reasons for this: first, in terms of database selection, we not only selected four English comprehensive databases, PubMed, Web of Science, EMBASE, and PsycINFO which are representative, but also four representative databases in China were also selected. So there is more than half of research come from China. Second, China is the most populous country in the world. NSSI among adolescents has become one of the most important public health problems in China, with more and more research input and published results, more and more Chinese studies are included in meta-analysis. In this way, the final summary of research results can be more comprehensive. Of course, due to the limitations of the author’s language, the lack of in-depth analysis of other related studies in French, German, Spanish, Japanese, Korean is also one of the limitations of this study. Second, the different studies used a wide variety of screening instruments and different cut-off points for NSSI, resulting in high heterogeneity among studies. Also contributing to heterogeneity were differences in study subjects, locations, and sociocultural environments. Lastly, we cannot ignore the risk of bias due to the self-report nature of NSSI instruments, which for socially taboo topics such as NSSI and suicide may not always be fully reliable.

## Conclusion

In summary, the global prevalence rate of NSSI in adolescents is high. Psychological, cognitive behavioral, family, and social interventions could be used to lower this number. Further research should be built on our findings and identify risk factors for self-harm in adolescents so that effective methods can be developed. With these actions, we can protect the health and safety of adolescents to the greatest extent possible. Administrators and the leaders of the community and hospital should create programs that teach adolescents how to deal with their emotions.

## Data availability statement

The original contributions presented in this study are included in the article/supplementary material, further inquiries can be directed to the corresponding author.

## Author contributions

QX and XS designed the study and developed the idea in consultation with LH, DH, and XH. XS and LH were responsible for literature screening. LH, DH, and XH extracted data. QX performed the statistical analyses. QX and XS drafted the manuscript and XH revised it. All authors read and agreed to the published version of the manuscript.
